# Spliced Leader RNAs, Mitochondrial Gene Frameshifts and Multi-Protein Phylogeny Expand Support for the Genus *Perkinsus* as a Unique Group of Alveolates

**DOI:** 10.1371/journal.pone.0019933

**Published:** 2011-05-24

**Authors:** Huan Zhang, David A. Campbell, Nancy R. Sturm, Christopher F. Dungan, Senjie Lin

**Affiliations:** 1 Department of Marine Sciences, University of Connecticut, Groton, Connecticut, United States of America; 2 Department of Microbiology, Immunology and Molecular Genetics, David Geffen School of Medicine, University of California Los Angeles, Los Angeles, California, United States of America; 3 Maryland Department of Natural Resources, Cooperative Oxford Laboratory, Oxford, Maryland, United States of America; Newcastle University, United Kingdom

## Abstract

The genus *Perkinsus* occupies a precarious phylogenetic position. To gain a better understanding of the relationship between perkinsids, dinoflagellates and other alveolates, we analyzed the nuclear-encoded spliced-leader (SL) RNA and mitochondrial genes, intron prevalence, and multi-protein phylogenies. In contrast to the canonical 22-nt SL found in dinoflagellates (DinoSL), *P. marinus* has a shorter (21-nt) and a longer (22-nt) SL with slightly different sequences than DinoSL. The major SL RNA transcripts range in size between 80–83 nt in *P. marinus*, and ∼83 nt in *P. chesapeaki*, significantly larger than the typical ≤56-nt dinoflagellate SL RNA. In most of the phylogenetic trees based on 41 predicted protein sequences, *P. marinus* branched at the base of the dinoflagellate clade that included the ancient taxa *Oxyrrhis* and *Amoebophrya*, sister to the clade of apicomplexans, and in some cases clustered with apicomplexans as a sister to the dinoflagellate clade. Of 104 *Perkinsus* spp. genes examined 69.2% had introns, a higher intron prevalence than in dinoflagellates. Examination of *Perkinsus* spp. mitochondrial cytochrome B and cytochrome C oxidase subunit I genes and their cDNAs revealed no mRNA editing, but these transcripts can only be translated when frameshifts are introduced at every AGG and CCC codon as if AGGY codes for glycine and CCCCU for proline. These results, along with the presence of the numerous uncharacterized ‘marine alveolate group I' and *Perkinsus*-like lineages separating perkinsids from core dinoflagellates, expand support for the affiliation of the genus *Perkinsus* with an independent lineage (Perkinsozoa) positioned between the phyla of Apicomplexa and Dinoflagellata.

## Introduction

*Perkinsus marinus* is a pathogenic alveolate causing “dermo” disease in oysters in estuaries of the north and central American Atlantic and Gulf of Mexico coasts. Other species of *Perkinsus* cause similar afflictions in a wide variety of other marine molluscs worldwide, all of which yield serious losses for shellfish industries [1]. This genus belongs to the crown group of eukaryotes known as Alveolata, but its exact phylogenetic position is debated. Based on the presence of cell surface micropores and an apical complex, *P. marinus* is historically considered to be a member of the Apicomplexa (for review see [2]), an exclusively parasitic lineage responsible for malaria and other infectious diseases in humans and animals. However, *P. marinus* shares cytological features with dinoflagellates, such as flagellar spurs and closed mitosis [2]. Phylogenetic studies based on small subunit ribosomal RNA (18S rDNA) and some conserved proteins such as actin and tubulin also conclude that *P. marinus* is closer to dinoflagellates than to apicomplexans (e.g. review by [2]–[4]), and thus are an early branch of dinoflagellate [4], [5]. These results challenge a proposition that both *Perkinsus* spp. and related *Parvilucifera* spp. parasites should constitute an independent phylum named Perkinsozoa [6], [7].

Since spliced-leader (SL) *trans*-splicing occurs throughout the phylum Dinoflagellata (e.g. [8]–[11]) yet has not been found in apicomplexans and ciliates, SL *trans*-splicing appears to be unique to the dinoflagellates within the Alveolata [12]. Under this scenario, the presence of SL *trans*-splicing in *Perkinsus* spp. [8], [13] allies *Perkinsus* spp. with dinoflagellates. While dinoflagellates use a 22-nt conserved SL (DinoSL), *P. marinus* harbors a longer (22 nt) and a shorter (21 nt) SL, with sequences varying slightly from the canonical DinoSL [14]. In addition, the genome of *P. marinus* (∼86 million base pairs; Project ID: 12736, http://www.ncbi.nlm.nih.gov/genomeprj/46451) is closer in overall size to apicomplexans (9–60 million base pairs; http://www.ncbi.nlm.nih.gov/genomeprj), but orders of magnitude smaller than dinoflagellates (3–250 billion base pairs; e.g. [15], [16]), and *P. atlanticus* chromosomes are more like typical eukaryotic chromosomes than dinokaryotic chromosomes [17]. Thus, whether *Perkinsus* spp. should be considered dinoflagellates remains unresolved.

Mitochondrial (mt) mRNA editing is a distinct characteristic of dinoflagellates within Alveolata and can be a useful marker to assess whether a lineage of alveolate is a dinoflagellate [12]. RNA editing is a sequence re-tailoring process that can be recognized by changes in an RNA sequence compared to that of its encoding DNA. Initially used to describe the insertion or deletion of uridine residues in mitochondrial (mt) transcripts in the kinetoplastid protozoans, the term “RNA editing” now also refers to nucleotide (nt) substitutions in RNA that occur in a wide variety of organisms (see [18], [19] for review). In Alveolata, mt gene mRNA editing only occurs in dinoflagellates, displaying the greatest diversity of modifications yet described in the context of a single genomic environment. The frequency of editing events decreases from high levels in the late-branching lineages to none in the ancient lineages such as *Oxyrrhis* and *Noctiluca* (e.g. [12], [19]). It is unclear if *Perkinsus* spp. mt gene mRNAs undergo editing, but the mt *cox1* of *P. marinus* is not translatable by the standard or mitochondrial codon table. The reading frame must be shifted 10 times by an unknown mechanism to yield a consensus COX1 protein [20]. Once verified, this bizarre process may be used as another molecular feature to demarcate *Perkinsus* spp. from dinoflagellates.

In this study we investigated the *Perkinsus* genus for the SL RNA gene structure, intron prevalence, full-length mt *cox1* and cytochrome *b* (*cob*) genes and their transcripts, and multiple-protein phylogenetic position. With the help of the GenBank database for six species and 33 unidentified *Perkinsus* sp. strains, and the *P. marinus* genome sequence, we performed thorough phylogenetic analyses and identification of introns in *P. marinus*. We paid special attention to histones because these proteins were thought to be absent in dinoflagellates until recently (for review see [21]). We used deduced full-length amino acid (aa) sequences of 41 genes to reconstruct phylogenetic trees. Genomic structures and corresponding RNA sequences of the SL gene were analyzed. Sixty-eight *Perkinsus* full-length cDNAs obtained in our previous studies [8], [14] were mapped to genome sequences to identify corresponding genes, and combined with 36 other reported genes to determine the frequency of introns. Although the *Perkinsus* clade shares commonalities with dinoflagellates, our data show that it is a unique lineage basal to the monophyletic clade of dinoflagellates.

## Materials and Methods

### *Perkinsus marinus* and dinoflagellate cultures, RNA isolation and cDNA construction

*Perkinsus marinus* isolate ATCC 50439 and *P. chesapeaki* ATCC PRA-65 were grown in tissue culture flasks with liquid media, samples (3–4×10^6^ cells) were collected by centrifugation and total RNAs were isolated as reported previously [14]. Dinoflagellates *Amphidinium carterae* (CCMP1314) and *Karlodinium veneficum* (CCMP2778) were grown in f/2 seawater medium at 20°C at a 12 h∶12 h light∶dark photocycle with a photon flux of approximately 50 µE·m^−2^s^−1^. When the cultures were in the exponential growth phase, ∼1×10^6^ cells were harvested and total RNAs isolated according to Zhang et al. [8]. These RNAs were used for cDNA synthesis as described previously [8].

### Identification of the SL RNA genes from the *P. marinus* genome project

*Perkinsus* spp. were suspected to possess a SL sequence similar to that of dinoflagellates (DinoSL; [8]). Two types of SL sequences were detected at the 5′ end of *P. marinus* full-length cDNAs of *pcna* and *cyclins* [14], PmaSL1, 5′-ACCGTAGCCATCTTGGCTCAAG-3′ (22 nt) and PmaSL2, 5′-ACCGTAGCCATCTGGCTCAAG-3′ (21 nt). These two *Perkinsus* SL sequences were used to query *P. marinus* whole-genome shotgun reads [http://www.ncbi.nlm.nih.gov/genomeprj/46451] to identify SL RNA genes. For hits with 85–100% identity to the queries, the genome sequences were collected for alignment with one another and with SL RNAs from dinoflagellates. Type-specific primers were designed for amplifying the putative SL RNAs (Table 1).

**Table 1:** SL RNA-related oligonucleotides used in this study.

Primer name	Sequence (5′-3′)	Application; reference*
dinoSLa/s	TGTACCTTGAGCCAAAATG	dinoflagellate SL RNA detection; [8]
PmaSL-La/s	CTTGAGCCAAGATGGCTACG	*Perkinsus* L-type SL RNA detection
PmaSL-Sa/s	ACCTTGAGCCAGATGGCTAC	*Perkinsus* S-type SL RNA detection
PmaSL-Li	TAGCGAGAGGACCTGATATC	*P. marinus* L-type SL RNA detection
PmaSL-Si	CAGAGAGYGGAMCTGATATT	*P. marinus* S-type SL RNA detection
LongSL1a/s	CCTCGCCGACATGCGGTGA	*P. marinus* L-type SL-like RNA detection
LongSL2a/s	TGGAGAGATGCGACCCAAA	*P. marinus* L-type SL-like RNA detection
shortSL1a/s	CGTCAACACGACTAGACATGA	*P. marinus* S-type SL-like RNA detection
shortSL2a/s	GCGACTAGTACTCATGTGAA	*P. marinus* S-type SL-like RNA detection
PmaSL-LS-F1	TGGCTCAAGGTARATATCAGKTCC	3′ RACE
PmaSL-LS-F2	CTCAAGGTARATATYAGKTCCDCYCKCT	3′ RACE
PmaSL-S2F2	CTCAAGGTAAATATCAGKTCCACTCT	3′ RACE
PmaSL-S2F3	AAGGTAAATATCAGKTCCACTCTCTG	3′ RACE
PmaSL-LNF2	CTCAAGGTAGATATCAGGTCCTCTCG	3′ RACE
PmaSL-LNF3	AAGGTAGATATCAGGTCCTCTCGCTA	3′ RACE
PmaSL-L1-like-F1	CCATCTTGGCTCAAGATCTTAGTG	3′ RACE
PmaSL-L1-like-F2	AGATCTTAGTGTCGTGTTATTGGTC	3′ RACE
PmaSL-L1-like-F3	GTGTTATTGGTCACCGCATGT	3′ RACE
PmaSL-L1-like-F4	GTTATTGGTCACCGCATGTCG	3′ RACE
PmaSL-L2-like-F1	GCCATCTTGGCTCAAGGTCTCA	3′ RACE
PmaSL-L2-like-F2	TTGGCTCAAGGTCTCAGTGTAC	3′ RACE
PmaSL-S-like-F1	GCCATCTGGCTCAAGTGTTG	3′ RACE
PmaSL-S-like-F2	CCATCTGGCTCAAGTGTTGTATT	3′ RACE
PmaSL-S-like-F3	TGTATTTTTGCTCCACATCATGTCTAG	3′ RACE
PmaSLS-like-F4	TTGCTCCACATCATGTCTAGTCGT	3′ RACE

*oligonucleotides from this study show no reference.

### RNA blot analyses of SL RNA

Total RNA from ∼10^6^ cells of both *Perkinsus* species and four strains of dinoflagellates in our previous studies [8], [9], including *Prorocentrum minimum* (CCMP696), *Polarella glacialis* (CCMP2088), *Karenia brevis* (CCMP2228) and *Karlodinium veneficum* (CCMP1975) were used for RNA blots. RNA samples were loaded onto an 8% acrylamide/8 M urea gel, a medium resolution gel optimal for RNAs below 350 nt, electrophoresed, and transferred to nylon membranes [22]. Oligonucleotide probes used for hybridization included dinoSLa/s for detection of the general dinoflagellate SL RNAs and the two types of *Perkinsus* SL RNAs (PmaSL-La/s and PmaSL-Sa/s hybridizing to exons and PmaSL-Li and PmaSL-Si to introns) (Table 1). The cDNA clones containing the two *P. marinus* SL RNAs were dot blotted to serve as positive controls for detection of the specific substrate SLs on RNA blots. Total RNA from *Leishmania tarentolae* cells was included to provide size markers. Oligonucleotide probes were labeled with γP^32^-ATP for hybridization [22].

### Rapid amplification of cDNA 3′ end (3′ RACE) and folding analysis

Poly (A) mRNA was depleted from *P. marinus* total RNA and a poly (A) tail was added to the remaining population using *Escherichia coli* Poly (A) Polymerase (Takara Mirus Bio) as reported [8]. First-strand cDNA synthesized using GeneRacer Oligo dT primer (Invitrogen) was used as PCR template. Two rounds of touch-down PCR were carried using the same conditions as above, with the extension time of 5 sec at 72°C. The first round of PCR was performed using PmaSL-LSF1 and GeneRacer3 primers. The PCR products were diluted 100-fold and used in the second round PCR with PmaSL-LSF2, PmaSL-LNF2, PmaSL-LNF3, PmaSL-S2F2, or PmaSL-S2F3, each paired with GeneRacer3, as the nested primers (Table 1).

Structures were modeled for the two dominant types of SL RNA transcripts using the MFOLD online program [http://mobyle.pasteur.fr/cgi-bin/MobylePortal/portal.py?form=mfold]. Folding was performed using the default setting except that the temperature was set at 27°C to match the *P. marinus* culture conditions.

### Mitochondrial gene analyses

The mt *cox1* and *cob* sequences were PCR-amplified from both genomic and cDNA templates using universal and *Perkinsus-*specific primers designed in this (Table 2) and previous studies [12], [23], [24]. PCR was performed with 30 cycles of 95°C for 15 sec, 50–58°C for 30 sec, and 72°C for 40 sec. PCR products were sequenced either directly or after cloning into a T-vector, with 5–10 clones randomly chosen for sequencing. To obtain the ends of the mt genes, we designed *Perkinsus*-specific primers for both *P. marinus* and *P. chesapeaki* (Table 2) based on the mt *cox1* and the partial *cob* sequences obtained from the newly released *P. marinus* genome shotgun sequence.

**Table 2:** Mitochondrial gene primers designed in this study.

Primer name	Sequence (5**′**-3**′**)	Application
Ncob5a	CAAATTATNACWGGWATWTTYTTRGC	Universal *cob* PCR
Ncob5c	GGTTAYGTNTTACCTTKDGGWCARATG	Universal *cob* PCR
Ncob5d	GGACAAATGTCTTWYTGGGSNGCNACNGT	Universal *cob* PCR
Ncob3a	GCRTARWANGGNTGRAARTACCAYTCNGG	Universal *cob* PCR
Ncob3b	TCYTGNGGRAAYTGNSCNCCDATCCA	Universal *cob* PCR
Ncob3c	GTTAGTAATNACWGTWGCWSCCCA	Universal *cob* PCR
PerkinsuscobF1	ATTAATGATAGTATTAATTATTTATGAAATATAAGG	*Perkinsus cob* PCR
PerkinsuscobF2	ACAAGTAATAACTTAGGCATAATAATAAAC	*Perkinsus cob* PCR
PerkinsuscobF3	GGTTTCATAGGTTATATATTAGGTTGG	*Perkinsus cob* PCR
PerkinsuscobF4	CATATTGGAGGTATAACAGTAATTATAAACT	*Perkinsus cob* PCR
PmacobNF1	GGTTATCGTTTATATACCCATAATTATACCC	*Perkinsus cob* PCR
PerkinsuscobNF2	TATATTACTAAGATATAATATCTAGTAAAGGGGA	*Perkinsus cob* PCR
PerkinsuscobR1	ACCTAGATATTAATAATATTAAATATGGTATGCCT	*Perkinsus cob* PCR
PerkinsuscobR2	AGTTTATAATTACTGTTATACCTCCAATATG	*Perkinsus cob* PCR
PerkinsuscobR3	CCAACCTAATATATAACCTATGAAACC	*Perkinsus cob* PCR
cox1_5a	TTATGATCTTCTTYWTNRTNATGCC	Universal *cox1* PCR
cox1_5b	GGAACAGGATGGACANTNTAYCCNCC	Universal *cox1* PCR
cox1_5c	TTCTGGTTCTTYGGNCAYCCYGARGT	Universal *cox1* PCR
cox1_3a	TAAACYTCRGGATGNCCRAADAACCA	Universal *cox1* PCR
Pmacox1F1	ATTGGTATATTAGGTATAGTATTATCTTAT	*P. marinus cox1* PCR
Pmacox1F2	AGAATACAATATAGGTACAGGCTGAA	*P. marinus cox1* PCR
Pmacox1R1	TTGAACCAATAGATGATATTAAATTCCA	*P. marinus cox1* PCR
Pmacox1R2	TGAACCAATAGATGATATTAAATTCCATAC	*P. marinus cox1* PCR
Perkinsuscox1NF1	TTCTATATATTAGTAAATAATAATAAAAGAATAGG	*Perkinsus cox1* PCR
Perkinsuscox1NF2	TTACATTAAATTATCAATAATTATTGGTATATTAGG	*Perkinsus cox1* PCR
Perkinsuscox1NF3	GTAAATTACTATAATATGGTTATAACATTACATGG	*Perkinsus cox1* PCR
Perkinsuscox1NR1	TTGATATTGAACCAATAGATGATATTAAATTCCA	*Perkinsus cox1* PCR
Perkinsuscox1NR2	ATAATATACCTAGCTAATAATGATATTACAGCACC	*Perkinsus cox1* PCR
Perkinsuscox1NR3	AATGGAAATGAGATACTATATAATATGTATCATGTAA	*Perkinsus cox1* PCR

### Generation of full-length gene sequences

Ribosomal proteins (RPs) from dinoflagellates [21] were used to query the *P. marinus* genome and GenBank databases to retrieve RPs from *P. marinus*, apicomplexans, ciliates, diatoms and other eukaryotic representatives. Since many of the dinoflagellate RP cDNAs available were not full-length, to maximize phylogenetic information from these genes, 22 full-length cDNAs of RPs from dinoflagellates *Amphidinium carterae* CCMP1314 and *K. veneficum* CCMP2778 were cloned using dinoflagellate-specific SL coupled with 3′ RACE as described previously ([8]; GenBank accession # GU372975-GU373034). To diversify the gene markers for phylogenetic analyses, another 12 conserved gene sequences were collected from our ongoing cDNA sequencing project for these two species, and their 5′ and 3′ ends achieved using RACE as necessary. Using these as queries, homologs were collected from GenBank for *P. marinus* and other species mentioned above. The absence of histones, long considered a benchmark of typical dinoflagellates, is erroneous (see [21] for review); thus, histone genes were retrieved from the *Perkinsus* genome project database. Full-length or nearly full-length mt *cox1* and *cob* sequences were also obtained from *P. marinus* and *P. chesapeaki*. The 3′ end of *cob* for both *Perkinsus* spp. was obtained using the 3′ RACE technique with *Perkinsus cob* primers paired with GeneRacer3 primer (Invitrogen). All of these genes were used in phylogenetic analyses.

### Multi-protein phylogenies

Predicted aa sequences of each gene were aligned with homologs from related organisms using CLUSTAL W (1.8) and inspected manually. Phylogenetic relationships of *P. marinus* with alveolate relatives and other eukaryotes were inferred using Neighbor Joining (NJ), Maximum Likelihood (ML), and MrBayes (MB) analyses. NJ analysis was performed online [http://clustalw.ddbj.nig.ac.jp/top-e.html] with the default setting. For ML tree reconstruction, the datasets were run through ProtTest [25] to identify the best-fitting aa substitution models (Table 3), which were then employed in the phylogenetic analysis using PhymLv3.0 [26]. MB analysis was carried out with 20,000–1,000,000 MCMC generations depending on when the average standard deviation of split frequencies reached below 0.01, a tree sampling frequency of 10–100, and 25% of generations discarded as burn-in [27]. To verify the reliability of the tree topologies, branch support was estimated based on bootstrap (1,000 resamplings) in NJ, approximate Likelihood Ratio Test (aLRT) in ML, and posterior probability in MB.

**Table 3:** Best substitution models selected by ProtTest for phylogenetic analyses.

Gene	Best Model	AIC	-lnL	Model parameters	
				alpha	p-inv
RPs concatenated	LG+I+G+F	1.00	56654	1.29	0.08
Other genes concatenated	LG+I+G+F	1.00	49591	0.63	0.15
Histone H2A	RtREV+I+G+F	0.99	6499	0.47	0
Histone H2B	LG+G+F	0.73	2440	0.59	0.12
Histone H3	LG+G	0.73	1625	0.73	0
Histone H4	JTT+G	0.58	917	0.58	0

### Analysis of Intron Frequency

Thirty-six and 37 unique full-length cDNAs from *P. marinus* and *P. chesapeaki*, respectively [8], were used as the queries to nBLAST-search against *P. marinus* genomic sequences to obtain the corresponding genomic DNA. The recently published full-length cDNAs and genomic DNAs for proliferating cell nuclear antigen (*pcna*) and two types of cyclins from *P. marinus* [14], as well as 36 other common protein-coding genes of *P. marinus* such as tubulins, *gapdh*, centrin, *hsp90* and ribosomal proteins reported in GenBank were compared (Table S1). Canonical GT/AG intron/exon boundaries validated the deduced intron start and end positions. The percentage of genes within this cohort that contained introns was determined.

## Results

### Two major types of *Perkinsus* SL RNA

**Figure 1:**
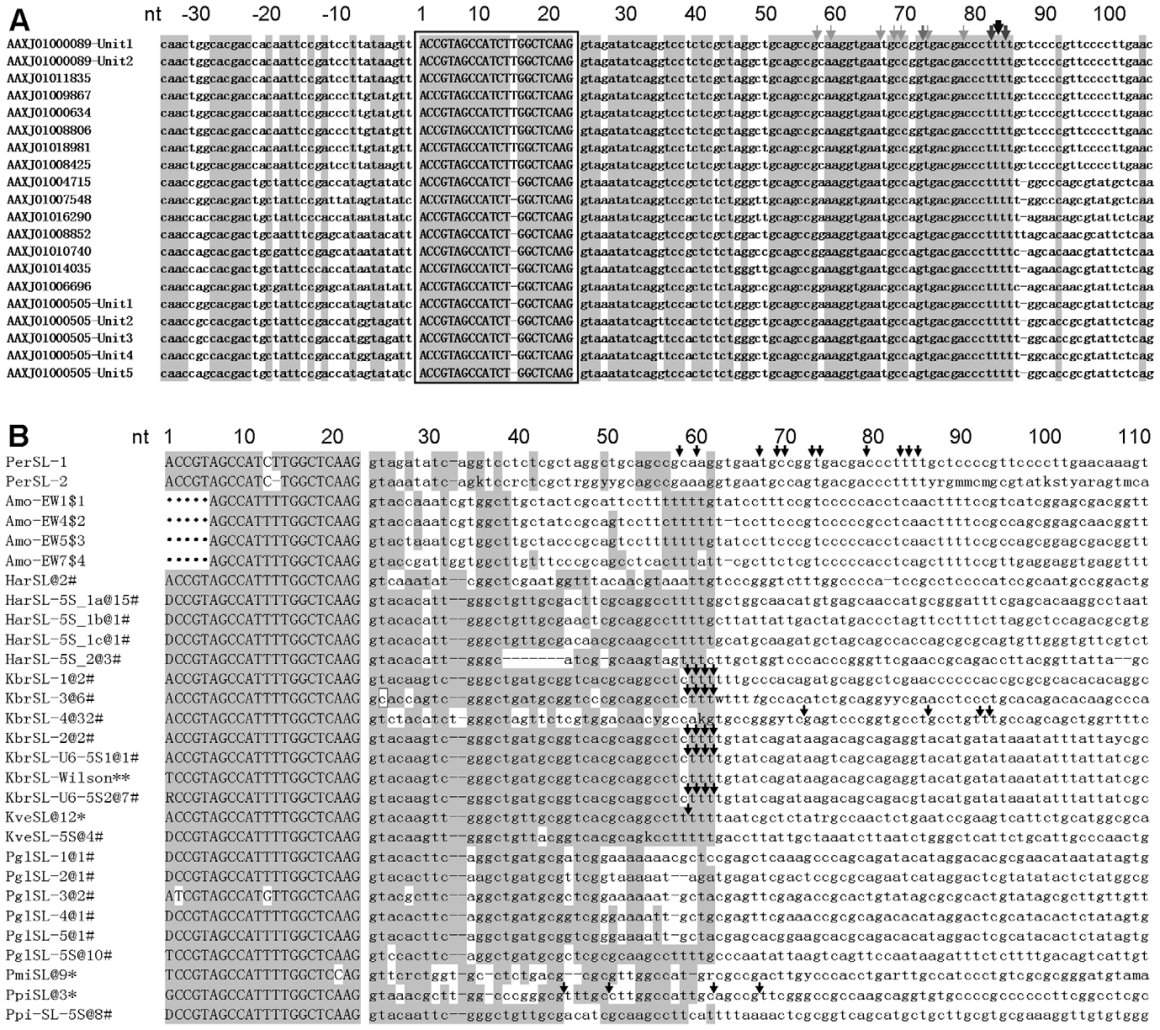
Alignments of spliced leader (SL) RNAs of *Perkinsus marinus* and dinoflagellates. A) Representative genomic sequences of two types of *P. marinus* SL RNA. B) *P. marinus* SL RNAs with the reported representatives of dinoflagellate SL RNA genomic sequences (modified according to [9]; the number of identical clones retrieved for each type is indicated by “@number” following the species abbreviation and type number). The SL region (boxed) is shown in uppercase letter, intron and the flanking regions are shown in lowercase letters, * indicates the conserved nucleotide (nt). The first ‘A’ of SL is numbered as nt 1. SL RNA transcripts mapped by 3′ RACE analyses are denoted by arrows to indicate the terminal positions, thickness with darkness of the arrows denote relative frequency of clones that ends where it is indicated. Note that the PCR-amplified *Amoebophrya* sp. genomic sequences contain only one unit of SL RNA gene, the partial SL sequence is of the primer used. Per, *P. marinus*, Amo, *Amoebophrya* sp.; Har, *Heterocapsa arctica*; Kbr, *Karenia brevis*; Kve, *Karlodinium veneficum*; Ppi, *Pfiesteria piscicida*; Pgl, *Polarella glacialis*; Pmi, *Prorocentrum minimum*. SL refers to SL RNA sequences obtained from SL-only repeats; SL-5S indicates SL RNA sequences from genes associated with 5S rRNA genes. *: sequences from [8]; **: sequence from [46]; #: sequences from [9], $1-4: GQ178071-GQ178074; •: sequences missing in the original reports. Shaded are conserved positions defined as identical in over six sequences in at least three species. A non-canonical C in the splice donor site of KbrSL-3 is boxed. Gaps introduced in the sequence alignment are shown as ‘–’.

From the reported *P. marinus* genome database we identified two major types of SL RNA genes: PmaSLRNA-L or L-type, and PmaSLRNA-S or S-type (Figure 1A), with the SL exons corresponding to the two SL sequences found previously in *pcna* and *cyclins* [14]. These sequences were similar to DinoSL (Figure 1B). For the L-type, we identified seven sequences (Table 3), and all but one (AAXJ01000089, containing two units of SL RNA) are 1–1.8 kb in length containing a single SL RNA gene. For the S-type, 42 sequences were identified with lengths ranging 1 to >14 kb (Table 4); of these, some were arrayed as tandem repeats or as a single unit clustered with both or either of the U2 and U4 snRNA genes downstream of the SL RNA gene; others were single or 2-unit tandem-repeat sequences not associated with U2 or U4 snRNA genes (Table 4).

**Table 4:** Genomic sequences containing SL RNA genes identified from *P. marinus* genome data.

Type	Accession number	No. of tandem repeats	Clustering with other genes
L-type	AAXJ01000089	2 units	None
	AAXJ01000634, AAXJ01008425, AAXJ01008806, AAXJ01009867, AAXJ01011835, AAXJ01018981	single unit	None
S-type	AAXJ01000505	5 units	U4, U2
	AAXJ01002433	2 units	U4, U2
	AAXJ01005490, AAXJ01006336, AAXJ01008092, AAXJ01007198, AAXJ01015199	single unit	U4, U2
	AAXJ01008392, AAXJ01008497, AAXJ01017256, AAXJ01001119, AAXJ01008189, AAXJ01014861, AAXJ01009920, AAXJ01007653, AAXJ01014749	single unit	U4
	AAXJ01000675, AAXJ01002387, AAXJ01010843	2 units	none
	AAXJ01006660, AAXJ01014035, AAXJ01016290, AAXJ01007696, AAXJ01004715, AAXJ01007548, AAXJ01005828, AAXJ01008852, AAXJ01009786, AAXJ01015286, AAXJ01006696, AAXJ01008101, AAXJ01010740, AAXJ01019396, AAXJ01007558, AAXJ01002161, AAXJ01006187, AAXJ01007317, AAXJ01003656, AAXJ01013911, AAXJ01009158, AAXJ01019701, AAXJ01015306	1 unit	none

### The major *P. marinus* SL RNA transcripts are 80–83 nt

The sequences containing the two types of *P. marinus* SL RNA genes (PmaSLRNAs) were conserved in the first 82–83 nt, with the SL exon of the L type 1-nt longer than that of the S type. Sequence similarity diminished in the downstream intron region. The sequence upstream of SL was more complex: for the L-type PmaSLRNAs, upstream sequences were uniform, whereas those of the S type were diverse, with some resembling the L type (Figure 1A). When PmaSLRNAs were aligned with the representatives of known dinoflagellate SL RNAs, PmaSLRNAs showed similarity in the exon (i.e. the 21/22-nt SL region) and moderate similarity in the beginning of the intron region (i.e., immediately downstream; Figure 1B). As in dinoflagellates, the predicted Sm-binding sequence was located in the SL exon of PmaSLRNAs, and the 3′ termini of the majority of substrate transcripts mapped within poly-T tracts, reminiscent of the termination element in SL RNAs of kinetoplastid [22], some dinoflagellates [9], and of other small RNA genes.

**Figure 2:**
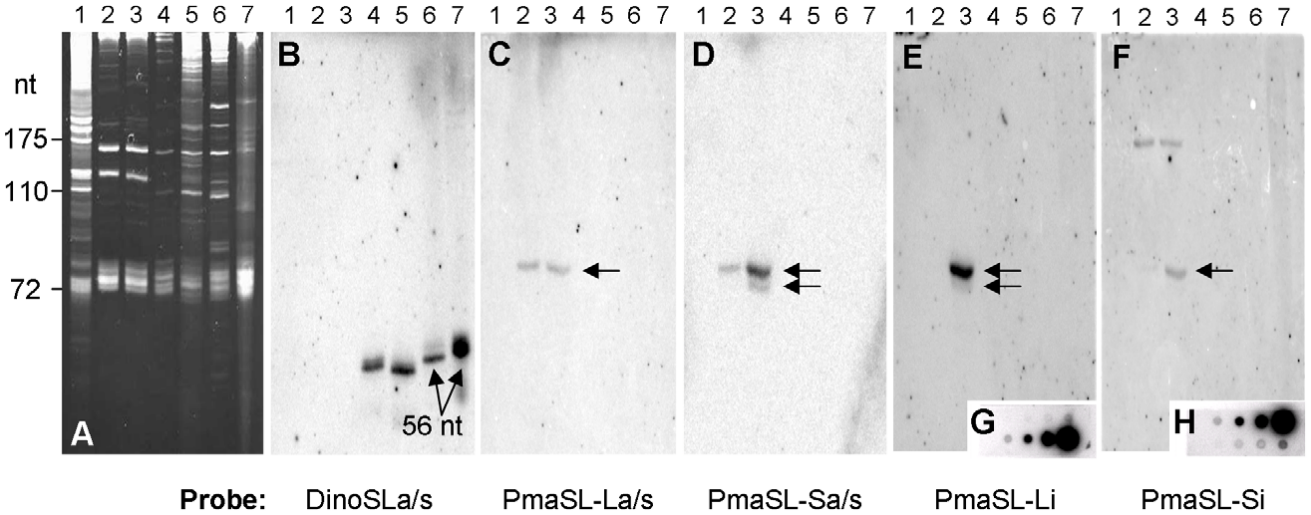
*Perkinsus* spp. substrate SL RNA larger than typical of dinoflagellates. A) Denaturing 8% polyacrylamide/8 M urea gel of total cell RNA from *Perkinsus* spp. and other organisms. Lane 1, *Leishmania tarentolae*; 2, *P. chesapeaki*; 3, *P. marinus*; 4, *Prorocentrum minimum*; 5, *Polarella glacialis*; 6, *Karenia brevis* and 7, *Karlodinium veneficum*. B–F) Probing of the blot shown in A) using oligonucleotides DinoSLa/s, PmaSL-La/s, PmaSL-Sa/s, and designed from intron regions in the PmaSL-L and PmaSL-S genotypes, respectively. Arrows highlight the SL RNA transcripts. G) and H) Dot blots of the PmaSLRNA-L (G) and PmaSLRNA-S (H) cDNA clones using the same probes as in (E) and (F), respectively.

The SL RNAs of two *Perkinsus* spp. and four dinoflagellates were analyzed by gel electrophoresis and hybridization. Ethidium bromide staining revealed that the two *Perkinsus* species have similar small RNA molecule profiles with commonalities to the dinoflagellate *P. minimum* (Figure 2A). Hybridization of an RNA blot of this gel with the 19-nt dinoflagellate SL probe DinoSLa/s (including 14 nt of SL and 5 nt of intron; Table 1) showed the dinoflagellate SL RNA pattern with major transcripts of <56 nt for the four dinoflagellates as reported previously [8], [9]; no hybridization was detected for the two *Perkinsus* species (Figure 2B). Probing the blot with *P. marinus* L-type or S-type SL probes (PmaSL-La/s and PmaSL-Sa/s respectively; Table 1), strong bands of >72 nt appeared in both *Perkinsus* species for both probes, with a minor band of slightly shorter length in the *P. marinus* sample for probe PmaSL-Sa/s; neither probe hybridized to dinoflagellate SL RNA (Figure 2C, 2D), indicating that the >72-nt bands are specific to the genus *Perkinsus*, and that *Perkinsus* SL RNAs are longer than those of typical dinoflagellates. Consistent with the similar RNA levels seen on the gel for the two *Perkinsus* species, probe PmaSL-La/s detected equivalent levels of this SL RNA variant (Figure 2C) in the two species. However, the band of *P. chesapeaki* was weaker than that of *P. marinus* with probe PmaSL-Sa/s (Figure 2D), possibly reflecting reduced expression or impaired hybridization due to a nucleotide alteration(s) in the exon region in *P. chesapeaki*. The minor band in the *P. marinus* lane may represent degraded SL RNA products. To further distinguish the two types of PmaSL RNA transcripts and to explore whether *P. chesapeaki* SL RNAs have similar introns to those of *P. marinus*, additional probes were designed for the PmaSLRNA L-type and S-type intron sequences (PmaSL-Li and PmaSL-Si; Table 1). Both intron probes revealed bands at >72 nt and some minor bands of <72 nt in *P. marinus* (Figure 2E, 2F), but no bands in *P. chesapeaki*, suggesting that *P. chesapeaki* SL RNAs have different intron sequences than *P. marinus*. An additional band appeared at ∼150 nt with PmaSL-Si for both *Perkinsus* spp. (Figure 2F), a likely result of non-specific hybridization to the abundant 5.8S ribosomal RNA (Figure 2A). To validate the specificity of the probes, 3′ RACE cDNA clones of the L- and S-type SL RNA were used to create dot blots that were hybridized separately with each probe. Each yielded a positive signal only when the corresponding probe was used (Figure 2G, 2H).

A 3′ RACE analysis gave an assortment of 3′ ends for both PmaSLRNAs. Of the 48 PmaSLRNA cDNA clones mapped, 25 ended at the 2^nd^ T, 11 clones at the 1^st^ T, and 4 clones ended at the 3^rd^ T of the poly-T tracts present in both SL genes, representing 83% of the ends obtained. Thus, most PmaSLRNA transcripts were 80–83 nt in length, corresponding to the major band observed in the RNA blots. The minor end classes of <72 nt may have contributed to the minor products seen by RNA blotting, possibly representing degraded or misprocessed SL RNA products.

### PmaSL present in protein coding genes and other genomic locations

BLAST analysis using PmaSL1 and PmaSL2 hit some cDNA or genomic DNA sequences apparently coding for proteins (e.g. EH076923, EH059894, EH059894, EH059894). In addition, over 100 genomic sequences were retrieved from the genome data that contained recognizable PmaSL1 (>60, e.g. AAXJ01000048, AAXJ01000335, AAXJ01000111, AAXJ0100359, AAXJ01004662, AAXJ01000077) and PmaSL2 (>40, e.g. AAXJ01000111, AAXJ01000162, AAXJ01000192, AAXJ01000237, AAXJ01000370) but no recognizable intron downstream. Most of these SL sequences started with T, and were arrayed in tandem repeats, and their downstream regions were variable. To investigate whether those SL RNA-like genomic sequences were also expressed, we designed primers (Table 1) containing a partial SL and downstream nucleotides or the downstream sequences alone and applied them to 3′ RACE and RNA blotting analyses. Neither of the approaches yielded clear products, indicating that these SL-like sequences are not functional SL RNA genes.

### Predicted PmaSLRNA structures and Sm-binding site locale

**Figure 3:**
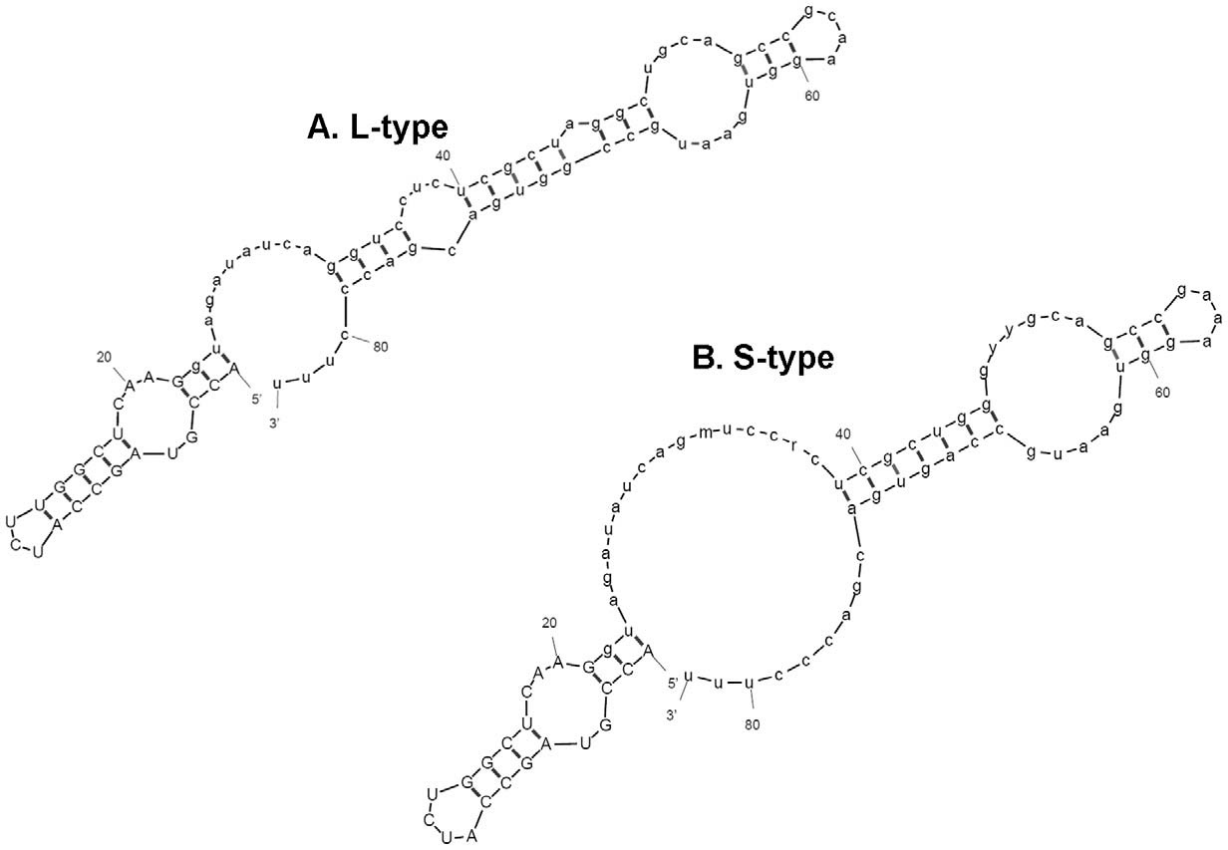
*Perkinsus marinus* SL RNA secondary structure similar to DinoSL RNA. Predicted structures of SL RNA for *P. marinus* L-type (A) and S-type (B) based on the most abundant cDNAs obtained. Model simulation was run using MFOLD: Prediction of RNA secondary structure modeling program (http://bioweb.pasteur.fr/seqanal/interfaces/mfold-simple.html) under default settings except that the folding temperature was set at 27°C, the culture temperature.

Similar to the situation in dinoflagellates, no apparent Sm-binding site sequence was found in the predicted intron regions of either of the PmaSLRNAs. Instead, AUUCUGG (L-type) or AUCUGG (S-type) found within the SL was the only recognizable candidate Sm-binding site, as in the DinoSL (AUUUUGG). The predicted intron region was similar between the two PmaSLRNAs, in contrast to the conserved intron in DinoSL RNAs, with the exception of the ancient parasitic genus of dinoflagellates *Amoebophrya* that showed considerable variation (Figure 1B). In the structural simulation using the default conditions for all but temperature, which was adjusted to the culture temperature of 27°C, the splice-donor dinucleotide (‘gu’ in ‘Gguag’) was double-stranded and the putative Sm-binding site (AUUCUGG/AUCUGG) single-stranded, forming a small terminal loop. The simulation yielded one comparable structure for both types of PmaSLRNAs (Figure 3). The predicted structures were similar to typical dinoflagellate SL RNA structures, having two stem-loops [8], [9], with the ‘extra’ intron region situated in a bulge of unpaired sequence connecting the two stem loops.

### Unique sequences and anomalous frameshifts in *Perkinsus* mt genes

**Figure 4:**
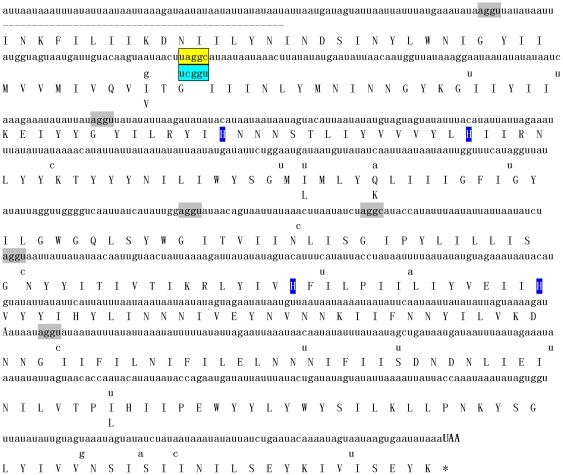
*Perkinsus* spp. *cob* and the predicted aa sequences. Sequences of *P. marinus*, and for *P. chesapeaki* only the sites with different nt/aa sequences, are shown. Four invariant His residues that are ligands for heme β are highlighted in blue. ‘-’ indicates missing sequence; the potential quadruplet codons ‘aggy’ for glycine are marked in grey, and quintuplet codons ‘uaggc’ and ‘ucggu’ for glycine are boxed.

All the possible combinations for *cob* primers designed based on dinoflagellate *cob* (Table 2; [12], [23], [24]) were tested but failed to PCR amplify any products. BLAST searching using *cob* aa sequences from apicomplexans and dinoflagellates against the *P. marinus* whole genome shotgun sequencing database (tblastx) hit one contig (860 bp, AAXJ01022806) containing the 5′ end of a *cob*-like sequence. The corresponding mRNA of this sequence and its 3′ end were obtained for both species of *Perkinsus* by PCR and 3′ RACE using *Perkinsus*-specific primers paired with the GeneRacer3 primer (GenBank accession numbers HQ670239, HQ670241; Figure 4, Table 2).

**Figure 5:**
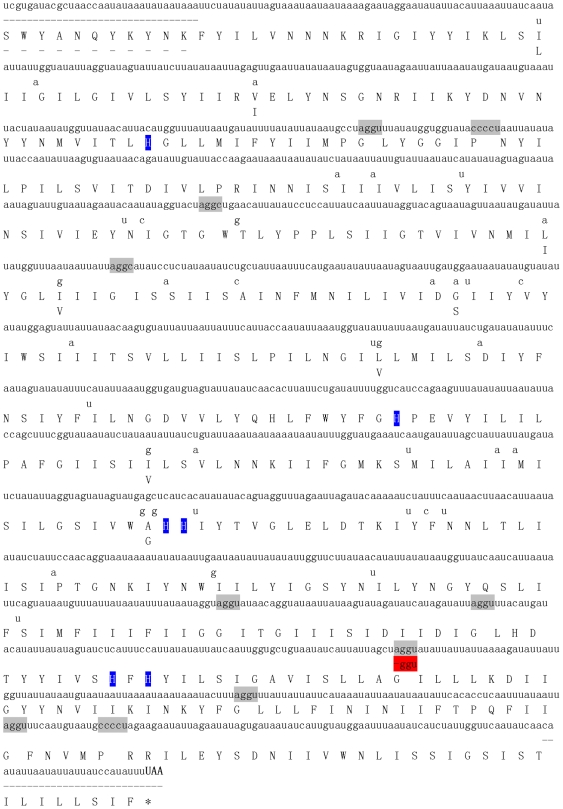
*Perkinsus* spp. *cox1* and the predicted aa sequences. Sequences of *P. marinus*, and for *P. chesapeaki* only the sites with different nt/aa sequences, are shown. Six invariant His residues that are ligands for heme α, Cu_B_ and heme α3 are highlighted in blue. ‘-’ indicates missing sequence; the potential quadruplet codons ‘aggy’ for glycine and ‘ccccu’ quintuplet codons for proline are marked in grey, and a standard ‘ggu’ codon for glycine in *P. chesapeaki cox1* is marked in red.

Using dinoflagellate *cox1* primer sets dinocox1F5-R3 [24] and universal *cox1* primer set cox1_5b-3a (Table 2), DNA fragments were amplified from genomic and cDNA templates of *P. marinus* (0.96 kb) and cDNA of *P. chesapeaki* (0.33 kb), respectively. Direct sequencing of these fragments proved that they were *cox1* sequences with 50–60% identity to that of dinoflagellates and apicomplexans. When the 0.96-kb *P. marinus cox1* sequence was used to BLAST against the *P. marinus* genome database, one 3147-bp sequence (AAXJ01004741) was obtained with 100% identity to the *P. marinus* DNA fragment we found. Nearly full-length cDNAs of *cox1* were generated by PCR amplification using *Perkinsus*-specific *cox1* primers for both *Perkinsus* species (GenBank accession numbers HQ670238, HQ670240; Figure 5, Table 2). Both the *cob* and *cox1* cDNA sequences matched the corresponding genomic DNAs, indicating that no mRNA editing events occurred in either transcript.

**Figure 6:**
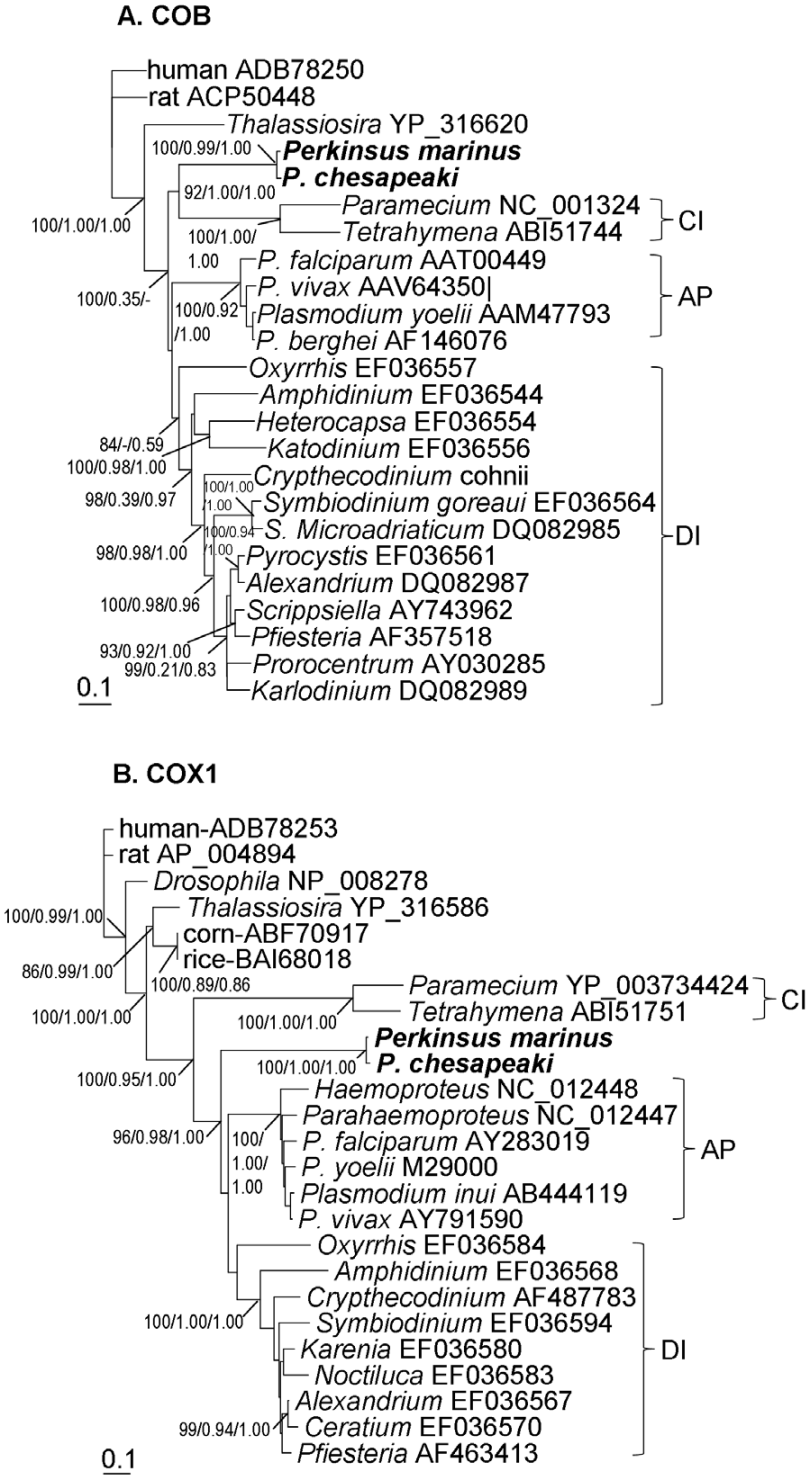
Phylogenetic affiliation of *P. marinus* with apicomplexans based on mitochondrial COB and COX1. The consensus trees with support from NJ (bootstrap, only >84% are shown), ML (aLRT), and MB (posterior probability). Brackets indicate clades of apicomplexans (AP), dinoflagellates (DI) and ciliates (CI).

Comparison of nt and deduced aa sequences of *Perkinsus cob* and *cox1* with counterparts in other alveolates revealed that correct translation of *Perkinsus* mt genes required the Mold/Protozoan/Coelenterate mt codon table (TGA codes for tryptophan) in general. To be fully translatable without internal stop codons, however, frameshifts had to be introduced at every AGG and CCC codon, the equivalent of using AGGY to code for glycine (six sites in *cob* and 7–8 sites in *cox1*) and CCCCU for proline (twice in *cox1*) (Figures 4, 5). An analogous result was reported by Masuda et al. [20] for the *P. marinus cox1*. Multiple cDNAs and genomic sequences substantiated these unusual reading frames, as well as the direct sequencing of PCR products. An interesting difference was found between the two *Perkinsus* species: at one site in *cox1,* glycine was encoded by an AGGU codon in *P. marinus*, but by a standard GGU codon in *P. chesapeaki* (Figure 5). With the introduction of these invoked anomalous quadruplet and quintuplet codons, the deduced aa sequences of the two *Perkinsus* COX1 were 98% identical to each other, 46–50% similar to the homologs in apicomplexans, 42–49% to dinoflagellates, 29–31% to ciliates, and 38–42% to other organisms (Figure 6). For *cob* (Figure 4), besides the quadruplet codon AGGY, glycine was also encoded by the quintuplet codons UAGGC (for *P. marinus*) and UCGGU (for *P. chesapeaki*). After these adjustments, the deduced COB aa sequences of the two *Perkinsus* spp. shared 97% similarity to each other, 34–36% to apicomplexans, 22–44% to dinoflagellates, 15–17% to ciliates, 27–33% to other organisms (Figure 6).

### High density of *cis*-introns relative to dinoflagellates

The corresponding *P. marinus* genomic sequences of 39 and 29 full-length cDNAs from *P. marinus* and *P. chesapeaki*, respectively [8], [14], were obtained. Comparison of these 68 cDNAs with the genomic DNA sequences revealed the presence of introns in 42 genes, yielding a 61.8% intron rate. Through GenBank database searches, we obtained an additional 36 common genes with known genomic structures, 30 of which have intron(s) (Table S1). Overall, the intron rate for *P. marinus* genes was 69.2% (72 out of 104). The intron-containing genes harbored between one and ten introns with the lengths ranging from 39 to 1622 bp, the majority of which were <100 bp.

### Multi-protein phylogeny of *Perkinsus* and other lineages

**Figure 7:**
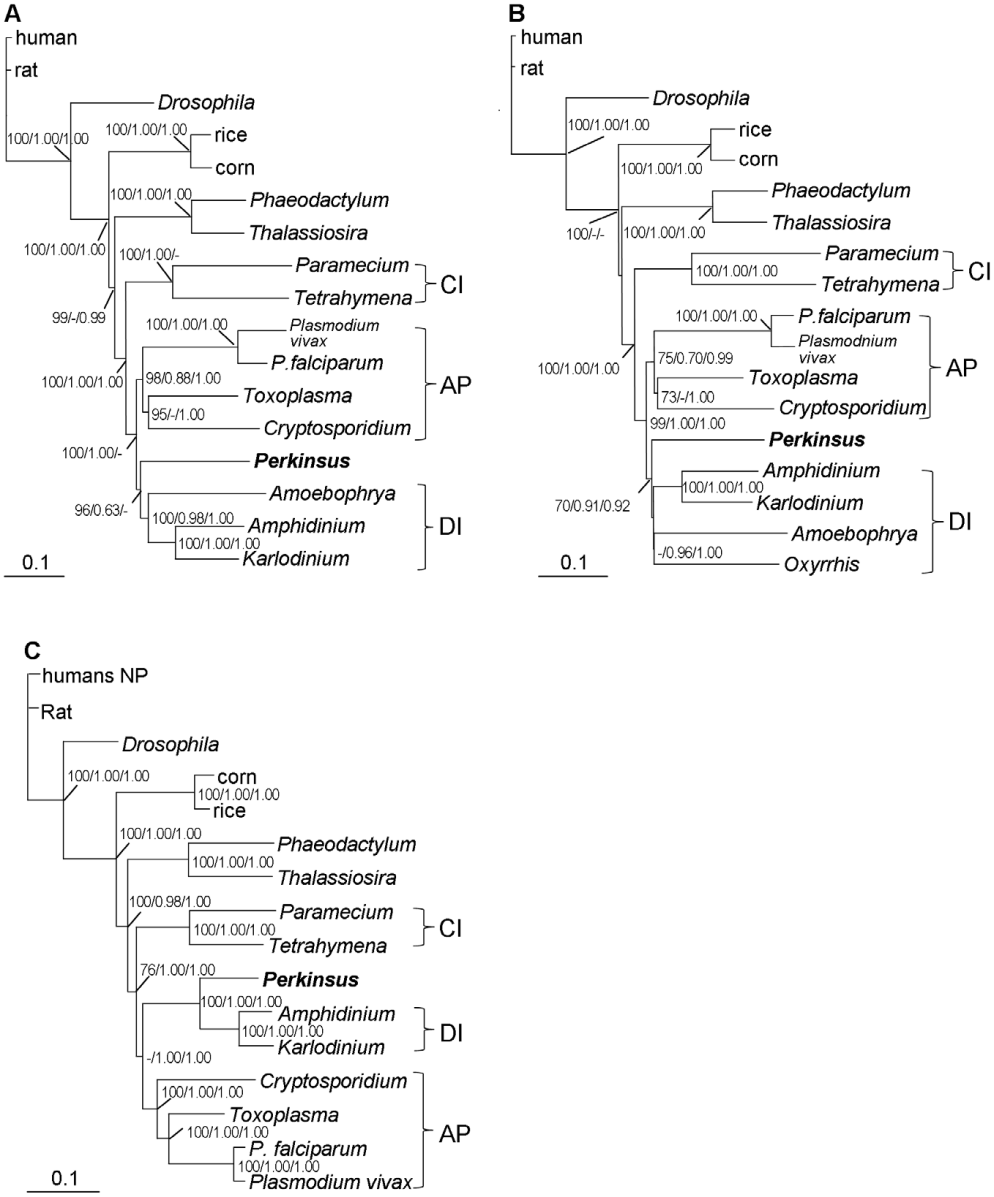
Phylogenetic affiliation of *P. marinus* with dinoflagellates and apicomplexans based on 30 conserved protein sequences. The consensus trees of concatenated genes of 19 ribosomal proteins (RPs) for 17 taxa (A), 8 RPs for 18 taxa including *Oxyrrhis* (B) and 11 non-RP proteins (C). Supports of nodes are from NJ (bootstrap), ML (aLRT), and MB (posterior probability). Brackets indicate clades of apicomplexans (AP), dinoflagellates (DI) and ciliates (CI).

Twenty-two ribosomal proteins were obtained for *Perkinsus* and various organisms; Maximum Likelihood (ML) trees inferred from the individual sequences gave varied tree topologies (Figures S1, S2, S3, S4). In general, *P. marinus*, dinoflagellates, apicomplexans, and ciliates formed a monophyletic group, while in several cases the heterokont diatoms, the closest relative of the alveolates, branched with some of the alveolate lineages, but without bootfostrap support. *Perkinsus* spp. allied with dinoflagellates in some cases (e.g. Figures S1C, 1F, S2E), and with apicomplexans (e.g. Figure S1D) or the diatoms (e.g. Figure S2D) in others, often with weak or no bootstrap support in these cases, indicating an unstable phylogenetic affinity. In contrast, NJ trees based on the 12 conserved protein sequences (actin, b-tubulin, GAPDH, α-tubulin, centrin, HSP90, EF1-α, ADP ribosylation factor, TIF5A, SmD1, cytochrome C and 14-3-3) produced similar tree topology, with *P. marinus* clustering with dinoflagellates in most of the cases (Figures S5, S6). For mt genes, *Perkinsus* spp. clustered with ciliates in COB tree, while allied with dinoflagellate/apicomplexan cluster in COX1 tree (Figure 6). When the concatenated RP sequence data (3,142 aa) was used, analyses using NJ, ML, and MB produced trees of similar topologies in which *P. marinus* branched at the base of the dinoflagellate clade (Figure 7). This was true for the analyses both without (Figure 7A) and with (Figure 7B) the ancient dinoflagellate lineage *Oxyrrhis marina*. The only exception was the MB tree in which *P. marinus* was allied with the clade of apicomplexans (Figure 7A). Similarly, when the other 12 protein sequences were concatenated (3,879 aa) the consensus tree inferred from the three algorithms showed the close relationship between *P. marinus* and dinoflagellates (Figure 7C). In most of these concatenated trees, the alliance of *P. marinus* and dinoflagellates was supported.

**Figure 8:**
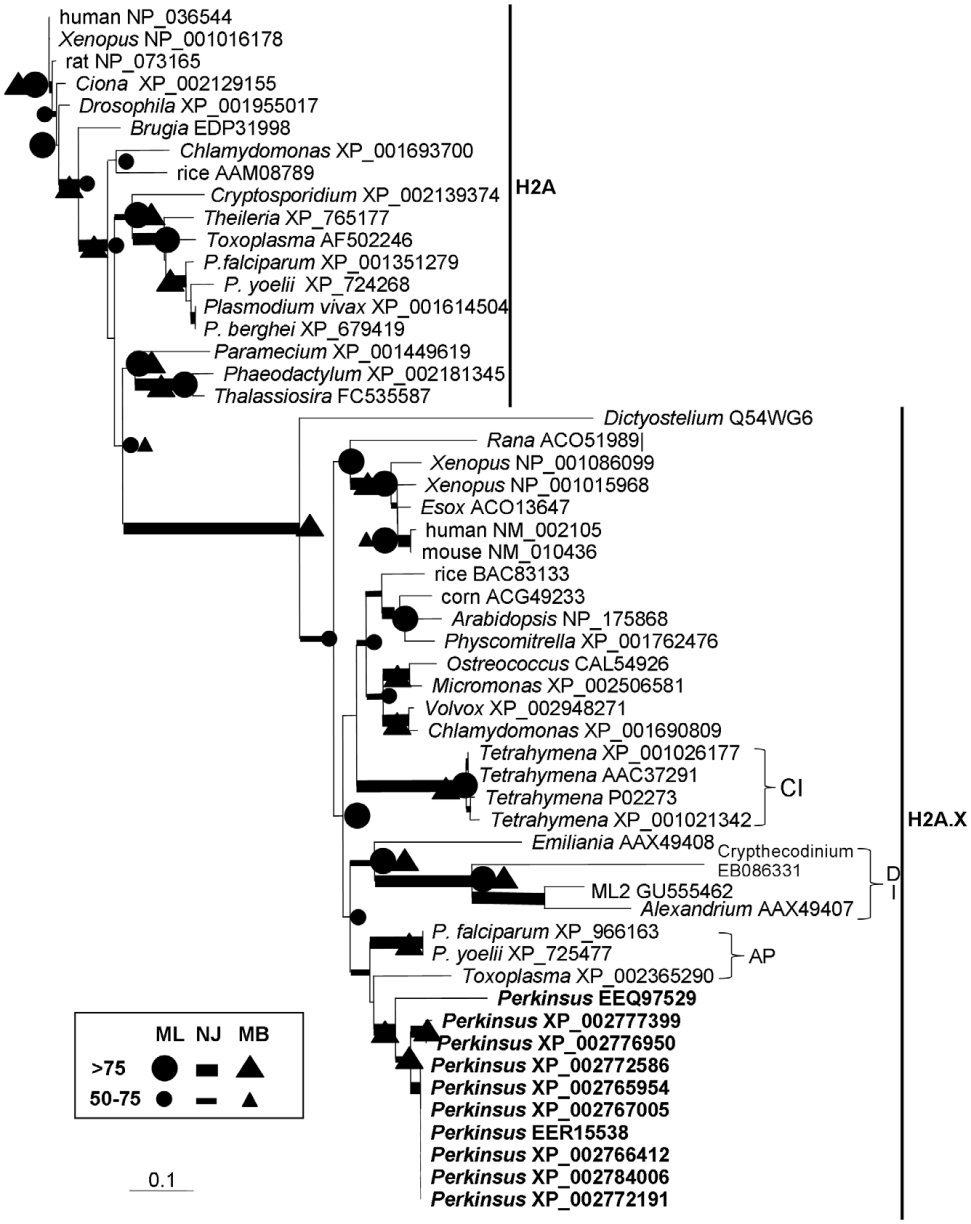
Phylogenetic affiliation of *P. marinus* with apicomplexans based on histone H2A. The canonical H2A and the isoform H2A.X consensus tree with support from NJ (bootstrap), ML (aLRT), and MB (posterior probability). Brackets indicate clades of apicomplexans (AP), dinoflagellates (DI) and ciliates (CI).

**Figure 9:**
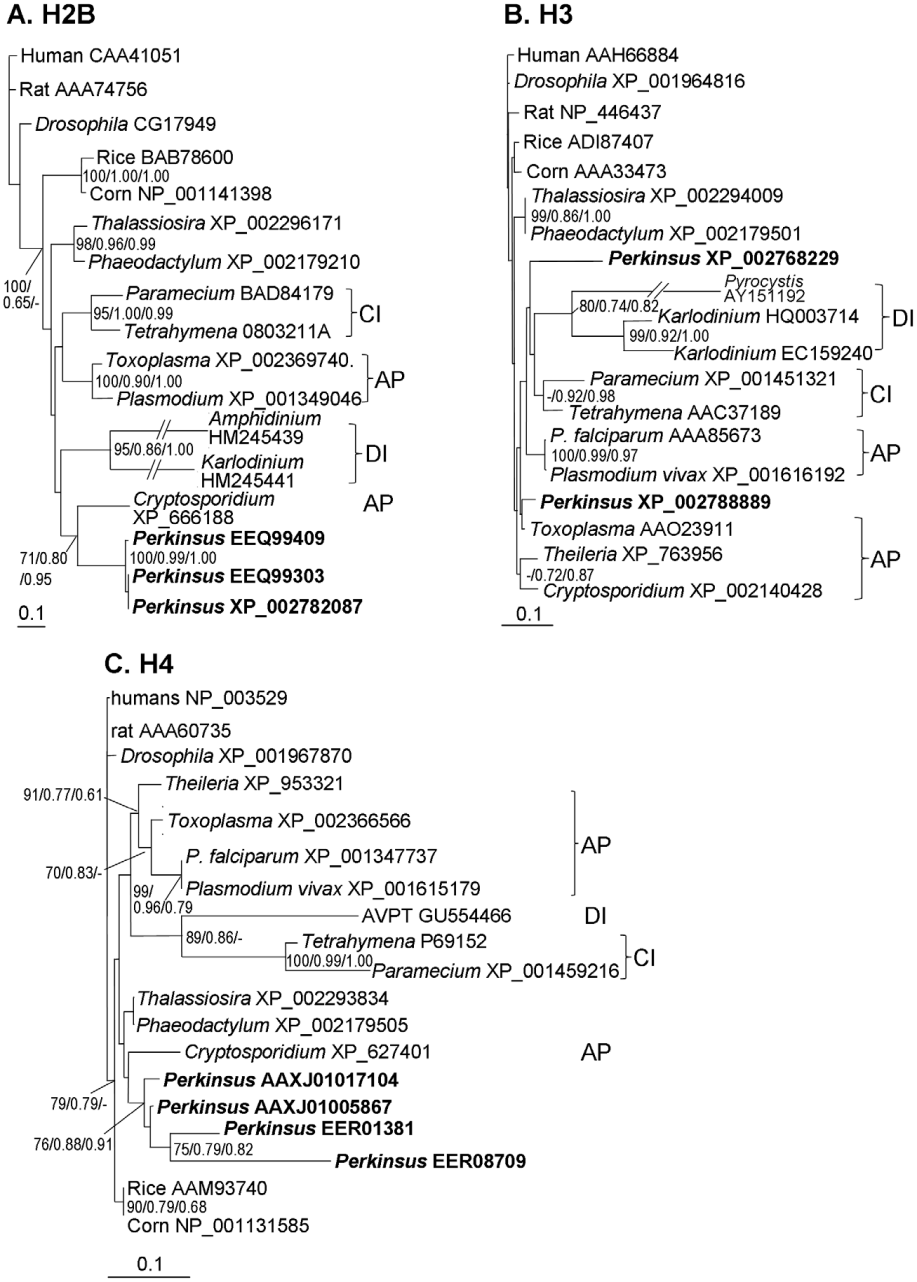
Phylogenetic affiliation of *Perkinsus marinus* with apicomplexan based on three histone proteins. Histone H2B(A), H3 (B), and H4 (C) consensus trees with support from NJ (bootstrap, only >70% are shown), ML (aLRT), and MB (posterior probability). Brackets indicate clades of apicomplexans (AP), dinoflagellates (DI) and ciliates (CI).

Multiple sequences were obtained for each of the *P. marinus* histones; in most of the phylogenetic trees, these sequences clustered together and allied with apicomplexans except for the H3 tree, in which one *P. marinus* H3 grouped with the apicomplexan *Toxoplasma gondii*, the other with dinoflagellate/ciliate clade (Figures 8, 9). Histone 2A in many organisms has acquired an isoform referred to as H2A.X. In both dinoflagellates and *P. marinus*, H2A.X seems to be the dominant, if not the only, form. The homolog retrieved from the *P. marinus* genome was clustered with H2A.X in the clade of apicomplexans (Figure 8).

## Discussion

To understand the evolution of parasitism in the Alveolata, the phylogenetic relationship among the major lineages in this crown group must be resolved accurately. No consensus exists for the relationship between the *Perkinsus* genus with other alveolates, particularly the partition between apicomplexans and dinoflagellates. Taking advantage of SL RNA, mt gene characteristics, gene structure (e.g. intron density), and the increasing availability of functional protein sequences, robust evidence is provided in support of a relatively close relationship between *Perkinsus* spp. and dinoflagellates, in addition to a distinct non-dinoflagellate position of this alveolate pathogen.

### *Perkinsus* SL RNAs mark earlier emergence and more complex evolution of *trans*-splicing in alveolates

PmaSLRNA sequences are similar to those of dinoflagellate SL RNAs, including the location of an apparent Sm-binding domain in the exon rather than in the intron, as is the case typically in other SL *trans*-splicing eukaryotes (see [8], [9] for review). The SL has left its footprints in other parts of the dinoflagellate genome in the form of single and tandem exon repeats located adjacent to the 5′ UTRs of protein coding genes [28]. This apparently unproductive phenomenon is postulated to occur when SL-containing mRNA are reverse-transcribed and integrated to the genome [28] but could also be a result of chromosome cross-over recombination [16]. Likewise, SL exons in single or multiple units were found in some *P. marinus* genes. The S-type SL with L-type intron was also suggested to exist based on PCR-amplified cDNA sequences of *P. marinus* SL RNA [29], although it requires verification by further genomic analysis.

PmaSLRNAs are distinct from dinoflagellate SL RNAs. In the apparent Sm-binding site, instead of a “TTTT” motif conserved in dinoflagellates, PmaSL has “TCTT” or “TCT”. The intron region of the SL RNA in dinoflagellates is conserved, but the similarities diminish in *Amoebophrya*, a parasitic lineage currently considered to represent the most ancient dinoflagellate [30]. SL RNAs in *P. marinus* display similar divergence from dinoflagellates, with a substantially longer intron relative to the core dinoflagellates and *Amoebophrya*, suggestive of an earlier divergence for *P. marinus*. The SL RNAs in other SL *trans*-splicing eukaryotes range from 46 nt in the urochordate *Ciona intestinalis* to 142 nt in *Trypanosoma brucei*. The SL RNA transcripts in *P. marinus* range from 80–83 nt, and are ∼83 nt in *P. chesapeaki*. Thus, *Perkinsus* SL RNAs have unique features in comparison to dinoflagellates, and *Perkinsus* spp. may represent the earliest *trans*-splicing lineage within Alveolata, separated from the non-*trans*-splicing Ciliophora and Apicomplexa [8], yet distinct from the Dinoflagellata. Given the high diversity of the parasitic Syndiniales class of dinoflagellates [31], the uncharacterized marine alveolate group I that lies between *Perkinsus* and the core dinoflagellates ([7] and references therein) should be examined for the presence of additional types of SL RNA.

### *Perkinsus* is a distinct pre-dinoflagellate taxon

As SL *trans*-splicing occurs in both basal (e.g. *Amoebophrya* and *Oxyrrhis*) and advanced (e.g. *Alexandrium*) lineages of dinoflagellates but not in apicomplexans and ciliates [8], the two closest relatives of dinoflagellates, the occurrence of this distinct mRNA processing mechanism is considered a defining indicator for dinoflagellates [12]. The presence of SL RNA *trans*-splicing in *Perkinsus* spp. indicates its inclusion in or alliance with the phylum of dinoflagellates, in accord with previous molecular phylogenetic studies (e.g. [2]–[4], [30]). Likewise, our multi-protein phylogenies consistently show that *P. marinus* is related to dinoflagellates among other representative eukaryotes with moderate-to-strong bootstrap support. Among the many single-gene phylogenetic trees, the majority is in agreement with the concatenated protein trees. In all trees, *P. marinus* was positioned as the earliest divergent even when *Oxyrrhis*, a genus hypothesized to be a pre-dinoflagellate [32] or an ancient lineage [12], was included. In addition, *P. marinus* was always placed basal to *Amoebophrya*, another ancient lineage of dinoflagellates.

Yet some degree of uncertainty exists in the phylogenetic position of *Perkinsus*. Contrary to the long-held notion that dinoflagellates did not possess nucleosomes and canonical histones, genes of all four major histones have recently been found in dinoflagellates (for review see [21]); however, dinoflagellate histones usually have unique sequences with insertions/deletions in several places, resulting long branches in the phylogenetic trees (Figures 8, 9). Comparing to dinoflagellates, *P. marinus* histones have typical eukaryotic sequences and group with apicomplexans in the phylogenetic trees. Besides histone trees, some other individual protein trees (Figures S1D, S2A, 2B, 2C, S3B, S5C) also show an alliance of *Perkinsus* spp. with apicomplexans, in agreement with earlier morphological and cytological studies [2]. In rare cases, *P. marinus* is clustered with diatoms, apparently because the protein sequence was too short to provide strong support of any topology.

The current analysis is limited in that only the sequences from one or two species of *Perkinsus* were available. *Perkinsus* appears more distant from apicomplexans than from dinoflagellates; however its generally close relationship with the clade of dinoflagellates could be due to the absence of taxa from intermediate lineages such as marine alveolate group I, additional taxa from the Perkinsozoa (e.g. *Parvilucifera* spp.), and dinoflagellates of the class Syndiniales.

*Cis*-splicing is thought to be uncommon in dinoflagellates [2]; however, only a few dinoflagellates have been examined for the presence of introns (e.g. form II Rubisco in *Symbiodinium* [33], luciferase C in *Pyrocystis lunula* [34]). We have examined more than 30 genes such as *pcna*, form II Rubisco, 14-3-3, and centrin for several dinoflagellates ([35], [36] and our unpubl. results), and did not find introns. A relatively high intron density for a dinoflagellate is found in *Amphidinium carterae*, in which a survey of 31 genes yields a 48% *cis*-splicing rate [37]. Our analysis of 104 *Perkinsus* genes yielded a 69.2% *cis*-splicing rate, a level contrasting those found in most dinoflagellates, and closer to the >50% level found in apicomplexans [38], [39].

The unique reading frame shifting and the lack of mRNA editing for mt genes again mark *P. marinus* as distinct from typical dinoflagellates. Both *P. marinus cob* and *cox1* mRNAs are identical to their genomic DNAs, indicating that no mRNA editing occurs to correct the frameshifts in these mt genes. Masuda et al. [20] reported the full-length mt *cox1* mRNA from *P. marinus*, showing that this mRNA was not translatable with standard codon usage, due to a reading frame that had to be shifted a total of 10 times at every AGG and CCC codon to yield a consensus COX1 protein. One or two sites of +1 frameshifting have been documented in animal mt genes (for review, see [40]), but such extensive +1 and +2 frameshifts are unique. In retroviruses, a –1 frameshift is corrected by tRNA back-slippage over homopolymeric codons adjacent to a local secondary structure that may include a pseudoknot (for review, see [41]). Masuda et al. [20] suggest two feasible mechanisms for the translational frameshifts in *Perkinsus*: a ribosomal frameshift in which stalled ribosomes skip the first bases of these codons (similar to the model hypothesized by Beckenbach et al. [42]), or specialized tRNAs recognizing non-triplet codons AGGY and CCCCU to code for glycine and proline, respectively. In this study, we add *cox1* for *P. chesapeaki* and *cob* sequences for *P. marinus* and *P. chesapeaki*, which share the unusual AGGY codon with *cox1* and use other unusual codons (UAGGC and UCGGU) to encode glycine as well. Specialized tRNAs in the *Perkinsus* mitochondrial system recognizing non-triplet AGGY and CCCCU codons, and likely UMGGY as well, may be more likely than the ribosomal frameshifting scenario, as naturally occurring tRNA mutants suppress +1 frameshifts *via* an extended anticodon loop in *Escherichia coli* (e.g. [43]), and quadruplet codons are used in protein mutagenesis [44].

The *Perkinsus* lineage is remarkably distinct from, while close to, dinoflagellates, and is most likely an independent lineage, supporting the postulate that *Perkinsus* spp., along with *Parvilucifera* spp., constitutes an independent phylum dubbed Perkinsozoa, the fourth phylum in Alveolata [6]. Although not addressed directly, a number of recent phylogenetic trees containing taxa from marine alveolate group I and *Perkinsus*-related parasitic alveolates such as *Parvilucifera* spp. reinforce grouping of *Perkinsus* spp. as an independent phylum [7], [45], [46]. Future phylogenies with broader taxon sampling that include species from *Parvilucifera* spp., Syndiniales in addition to *Amoebophrya*, and marine alveolate group I representatives will refine the phylogenetic relationships among *Perkinsus*, dinoflagellates, and other alveolates.

## Supporting Information

Figure S1 ML phylogenetic trees of six of the 22 ribosomal proteins. A, RPL11; B, RPL17; C, RPL18A; D, RPL18; E, RPL21; F, RPL22. Groupings of major clades are labeled on the right. DI, dinoflagellates; AP, apicomplexans; CI, ciliates; PE, *Perkinsus*.
(TIF)

Figure S2 ML phylogenetic trees of six of the 22 ribosomal proteins. A, RPL26; B, RPL32; C, RPL34; D, RPL35A; E, RPL44; F, RP_P1. Groupings of major clades are labeled on the right. DI, dinoflagellates; AP, apicomplexans; CI, ciliates; PE, *Perkinsus*.
(TIF)

Figure S3 ML phylogenetic trees of six of the 22 ribosomal proteins. A, RPS3a; B, RPS5; C, RPS7; D, RPS10; E, RPS11; F, RPS13. Groupings of major clades are labeled on the right. DI, dinoflagellates; AP, apicomplexans; CI, ciliates; PE, *Perkinsus*.
(TIF)

Figure S4 ML phylogenetic trees of four of the 22 ribosomal proteins. A, RPS17; B, RPS25; C, RPS26; D, RPS27a. Groupings of major clades are labeled on the right. DI, dinoflagellates; AP, apicomplexans; CI, ciliates; PE, *Perkinsus*.
(TIF)

Figure S5 NJ phylogenetic trees of six of the 12 non-RP proteins. A, actin; B, β-tubulin; C, GAPDH; D, α-tubulin; E, centrin; F, HSP90. Groupings of major clades are labeled on the right. DI, dinoflagellates; AP, apicomplexans; CI, ciliates; PE, *Perkinsus*.
(TIF)

Figure S6 NJ phylogenetic trees of six of the 12 non-RP proteins. A, EF1-α; B, ADP ribosylation factor; C, TIF5A; D, SmD1; E, cytochrome C; F, 14-3-3. Groupings of major clades are labeled on the right. DI, dinoflagellates; AP, apicomplexans; CI, ciliates; PE, *Perkinsus*.
(TIF)

Table S1 *cis-introns* in *Perkinsus marinus*.
(XLS)
