# Supplementary figures and images for: Spliced Leader RNAs, Mitochondrial Gene Frameshifts and Multi-Protein Phylogeny Expand Support for the Genus Perkinsus as a Unique Group of Alveolates
Source: PLoS One. 2011 May 24;6(5):e19933. doi: 10.1371/journal.pone.0019933 (PMC3101222; doi:10.1371/journal.pone.0019933)

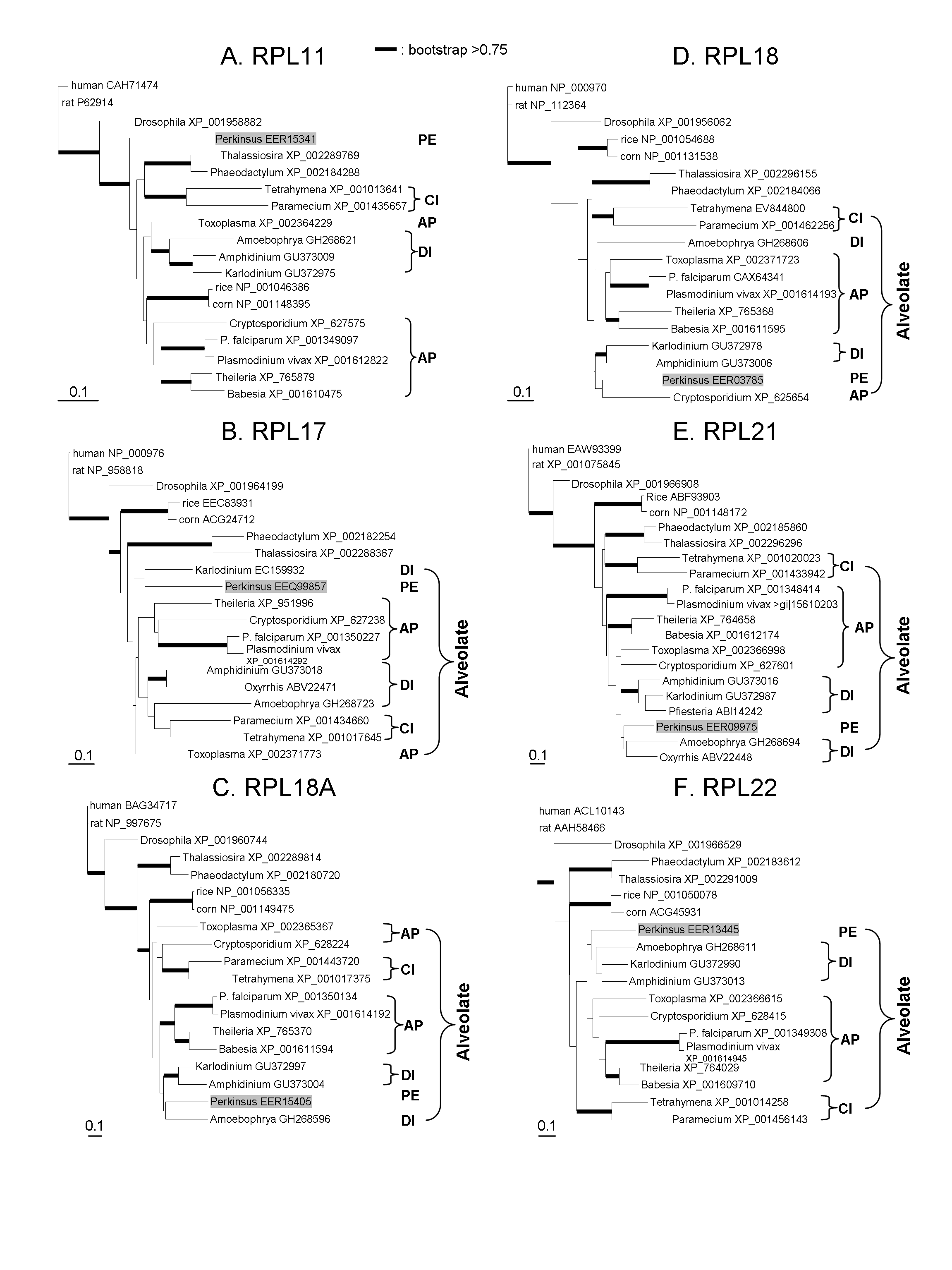

Supplement: Figure S1 — ML phylogenetic trees of six of the 22 ribosomal proteins. A, RPL11; B, RPL17; C, RPL18A; D, RPL18; E, RPL21; F, RPL22. Groupings of major clades are labeled on the right. DI, dinoflagellates; AP, apicomplexans; CI, ciliates; PE, Perkinsus. (TIF) [file pone.0019933.s001.tif]

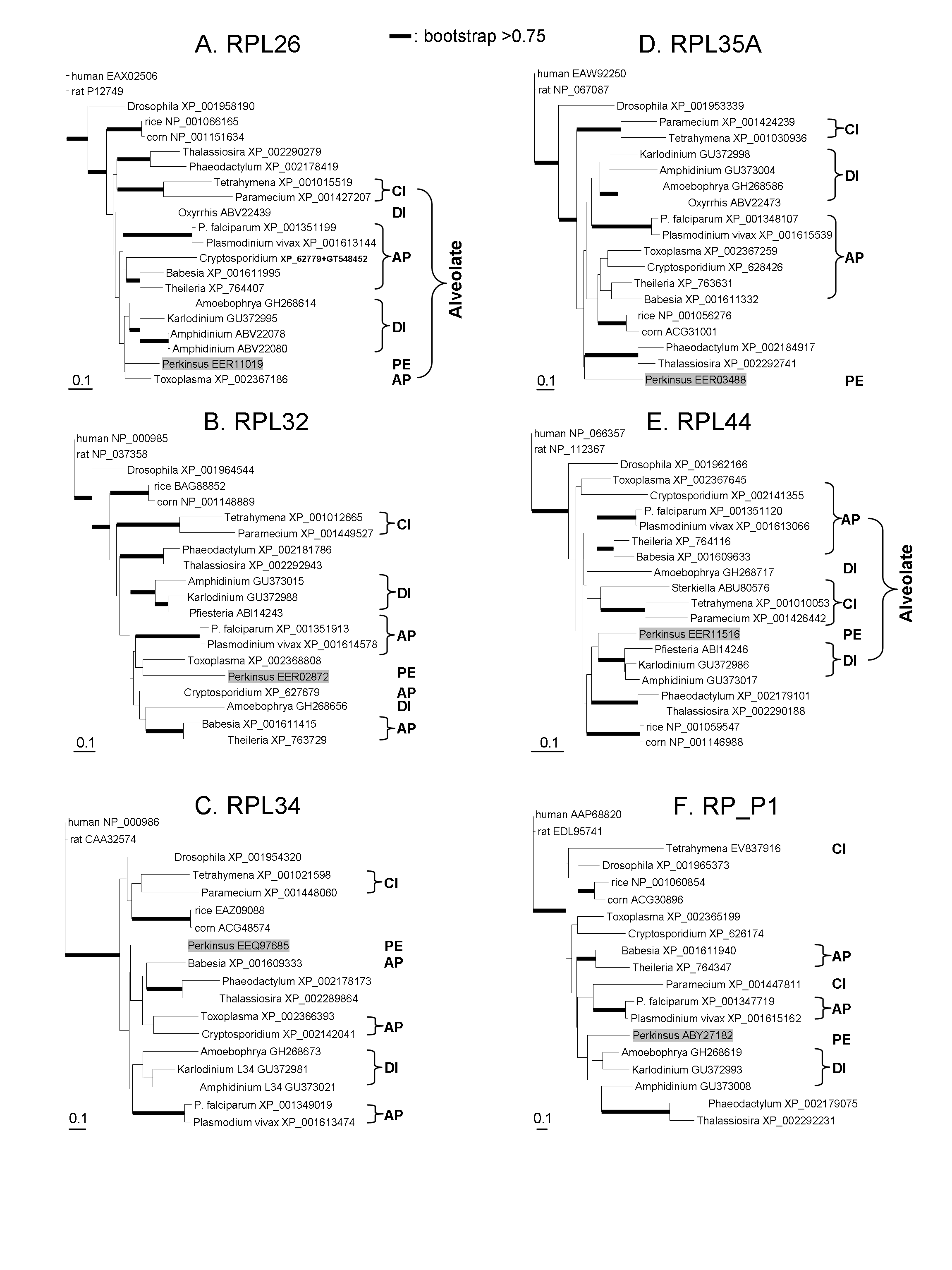

Supplement: Figure S2 — ML phylogenetic trees of six of the 22 ribosomal proteins. A, RPL26; B, RPL32; C, RPL34; D, RPL35A; E, RPL44; F, RP_P1. Groupings of major clades are labeled on the right. DI, dinoflagellates; AP, apicomplexans; CI, ciliates; PE, Perkinsus. (TIF) [file pone.0019933.s002.tif]

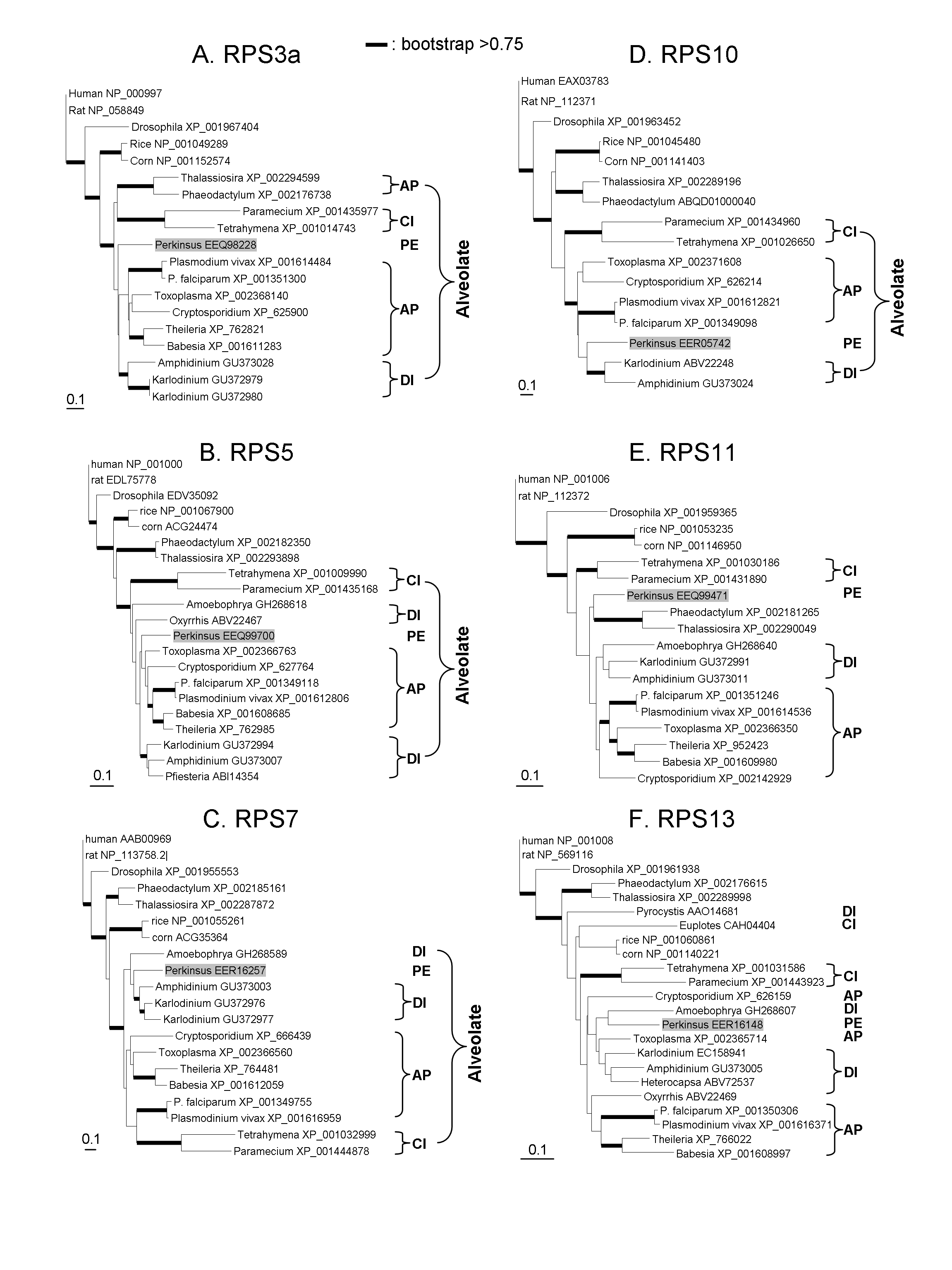

Supplement: Figure S3 — ML phylogenetic trees of six of the 22 ribosomal proteins. A, RPS3a; B, RPS5; C, RPS7; D, RPS10; E, RPS11; F, RPS13. Groupings of major clades are labeled on the right. DI, dinoflagellates; AP, apicomplexans; CI, ciliates; PE, Perkinsus. (TIF) [file pone.0019933.s003.tif]

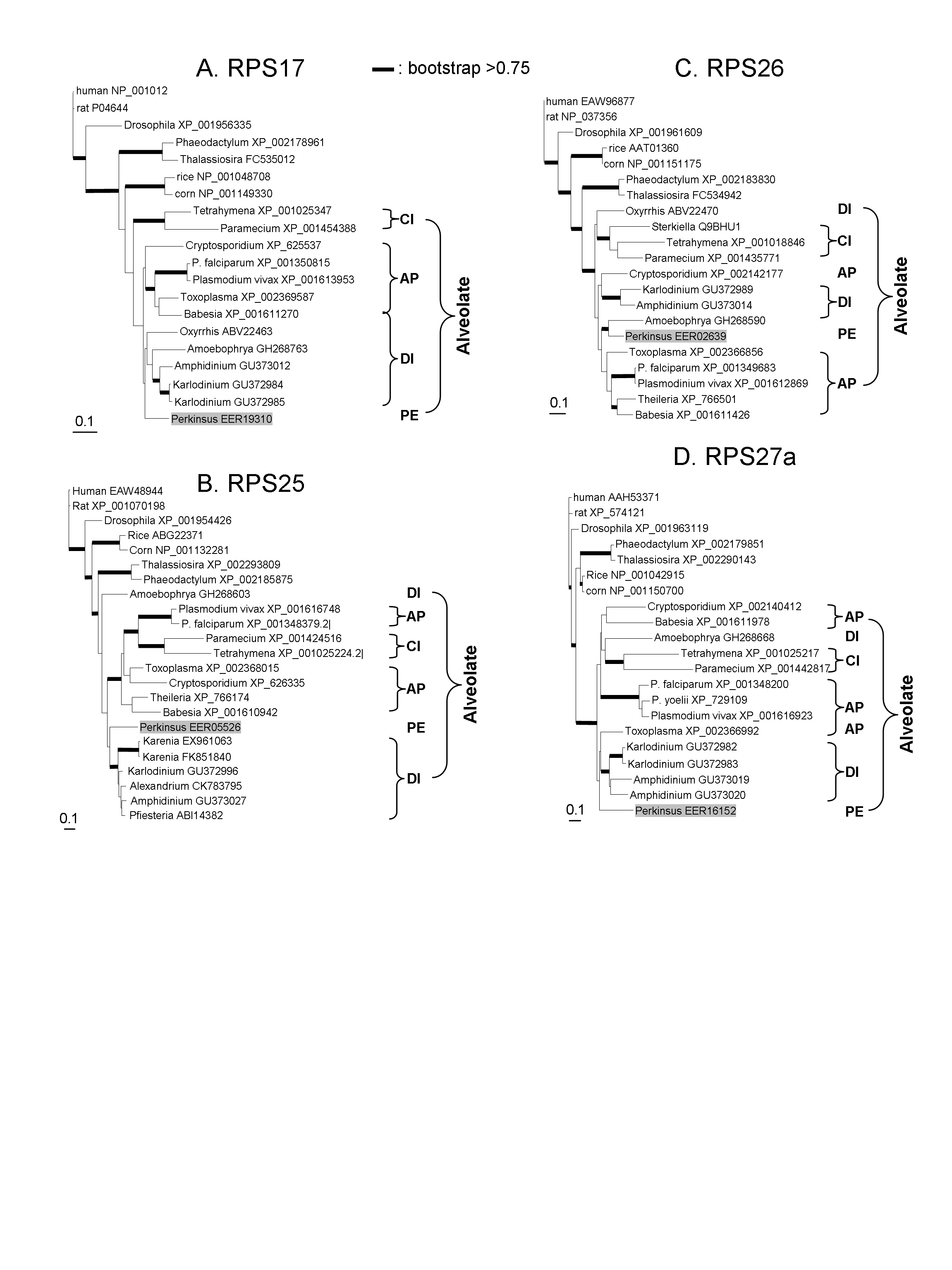

Supplement: Figure S4 — ML phylogenetic trees of four of the 22 ribosomal proteins. A, RPS17; B, RPS25; C, RPS26; D, RPS27a. Groupings of major clades are labeled on the right. DI, dinoflagellates; AP, apicomplexans; CI, ciliates; PE, Perkinsus. (TIF) [file pone.0019933.s004.tif]

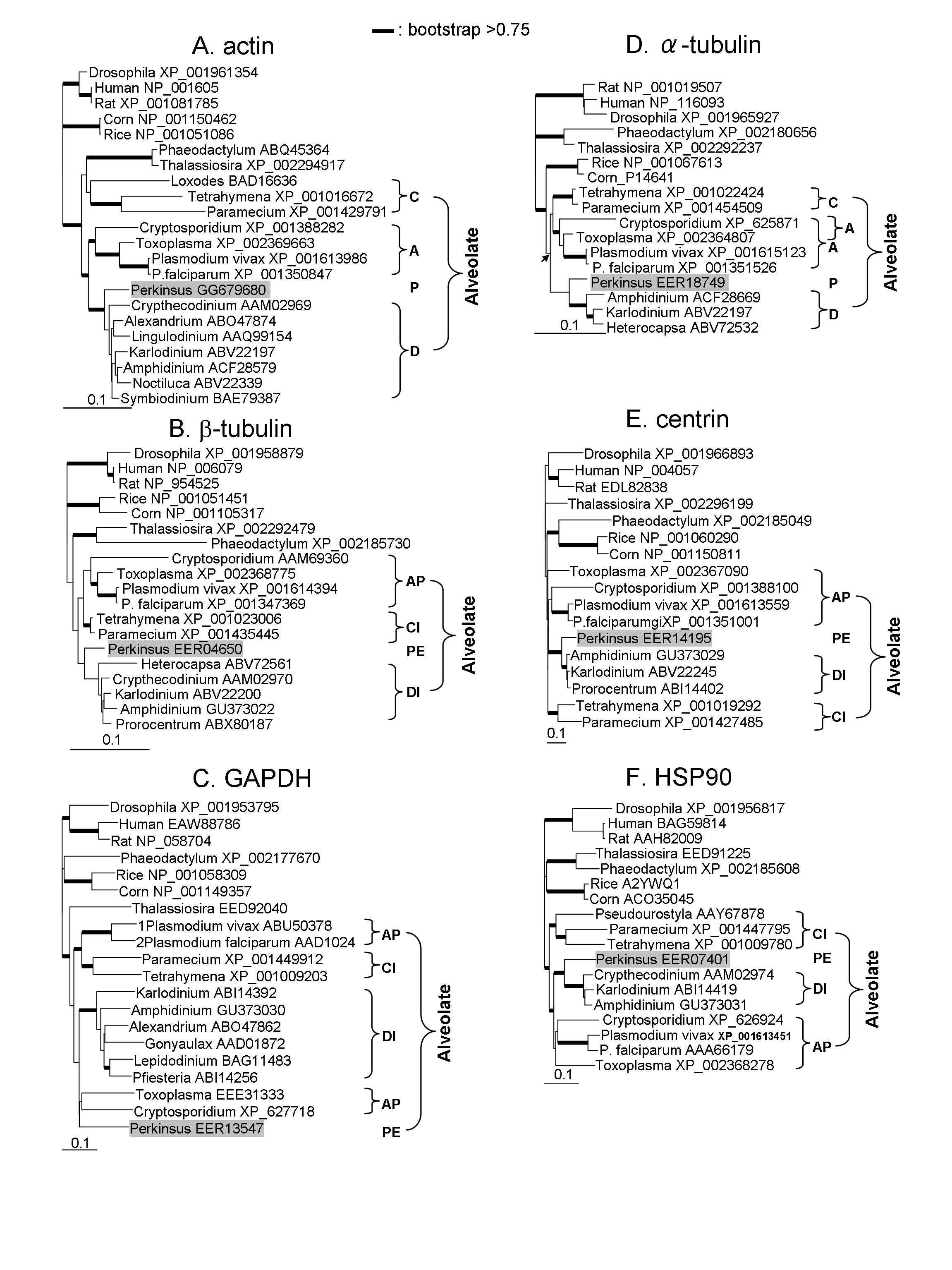

Supplement: Figure S5 — NJ phylogenetic trees of six of the 12 non-RP proteins. A, actin; B, β-tubulin; C, GAPDH; D, α-tubulin; E, centrin; F, HSP90. Groupings of major clades are labeled on the right. DI, dinoflagellates; AP, apicomplexans; CI, ciliates; PE, Perkinsus. (TIF) [file pone.0019933.s005.tif]

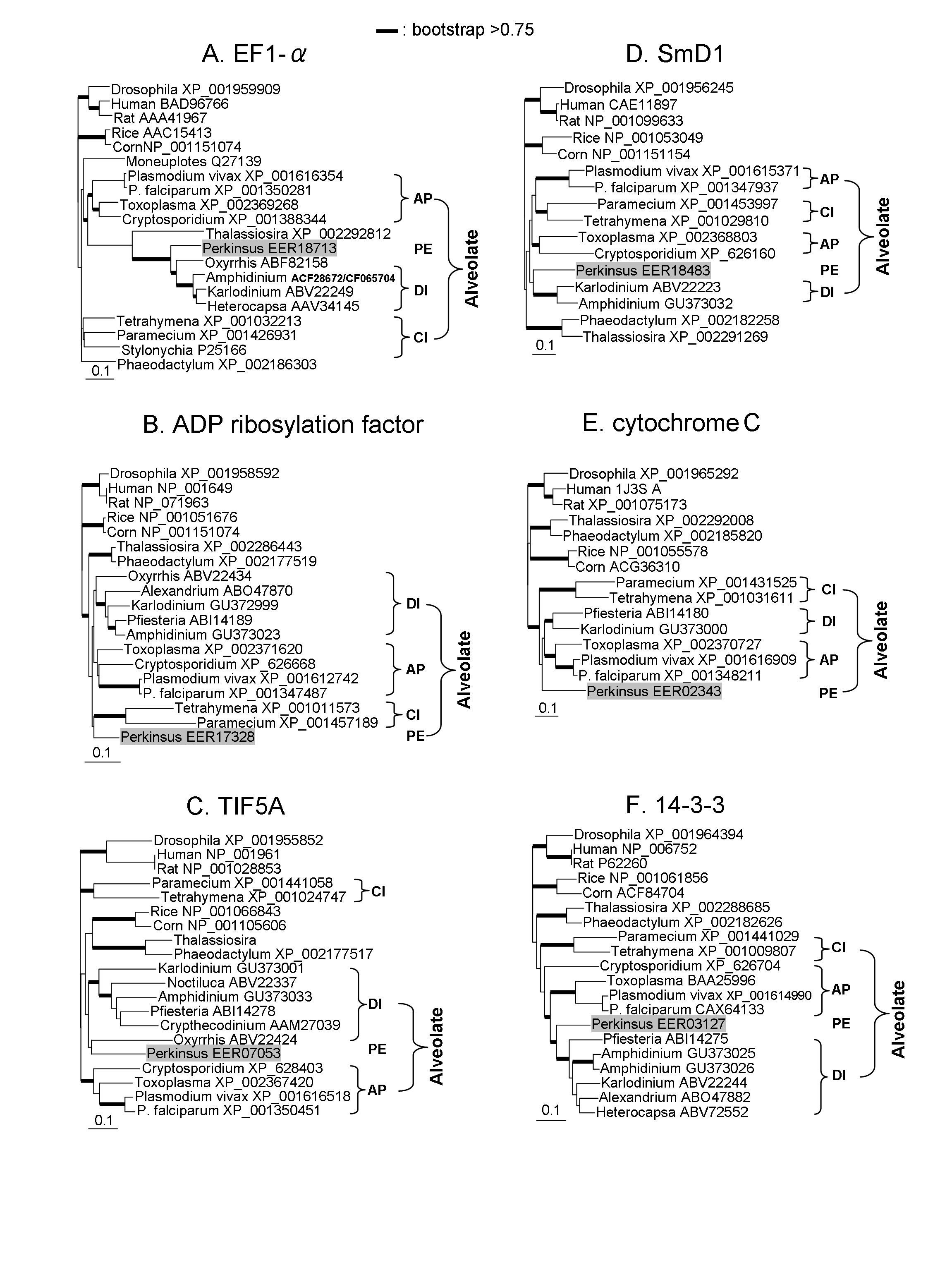

Supplement: Figure S6 — NJ phylogenetic trees of six of the 12 non-RP proteins. A, EF1-α; B, ADP ribosylation factor; C, TIF5A; D, SmD1; E, cytochrome C; F, 14-3-3. Groupings of major clades are labeled on the right. DI, dinoflagellates; AP, apicomplexans; CI, ciliates; PE, Perkinsus. (TIF) [file pone.0019933.s006.tif]
